# Intravenous Laser Blood Irradiation and Tocilizumab in a Patient with Juvenile Arthritis

**DOI:** 10.1155/2014/923496

**Published:** 2014-03-04

**Authors:** Dragos Andrei Chiran, Michael Weber, Laura Marinela Ailioaie, Eovelina Moraru, Constantin Ailioaie, Daniela Litscher, Gerhard Litscher

**Affiliations:** ^1^Faculty of Medicine, “Grigore T. Popa” University of Medicine and Pharmacy, 16 Universitatii Street, 700115 Iasi, Romania; ^2^Institute for Laser Therapy and Acupuncture, Sohnreystraße 6, 37697 Lauenförde, Germany; ^3^Department of Medical Physics, “Alexandru Ioan Cuza” University, 11 Carol I Boulevard, 700506 Iasi, Romania; ^4^Laser Clinic, 83 Arcu Street, 700135 Iasi, Romania; ^5^Second Pediatric Clinic, Street Mary Emergency Hospital for Children, 62 Vasile Lupu St., 700309 Iasi, Romania; ^6^Research Unit of Biomedical Engineering in Anesthesia and Intensive Care Medicine and TCM Research Center Graz, Medical University of Graz, Auenbruggerplatz 29, 8036 Graz, Austria

## Abstract

This study presents effects of intravenous laser blood irradiation (ILBI) in a transient immunodeficiency patient with juvenile idiopathic arthritis (JIA) treated with an interleukin-6 receptor inhibitor (Tocilizumab). Biological agents induce JIA remission, but some patients do not respond favorably to this final therapeutic line of defense. ILBI was performed in a 16-year-old male patient, with JIA and transient immunodeficiency. When ILBI was introduced, the patient was receiving disease-modifying drugs, steroids, tocilizumab, and physical therapy. Because the disease was not well controlled, ILBI was applied in addition to other ongoing therapies. The patient underwent 1 session daily, and 10 successive sessions per month, repeated every 3 months, for 7 months. Patient evaluation was performed before ILBI was started and at 3, 6, 9, and 12 months after ILBI initiation, using the ACR Pediatric response. The outcome was evaluated using Pediatric 50, 70, and 90 responses and compared to initial status, after 3, 6, 9, and 12 months. At the end of study, the titre of IgA and IgG levels returned to normal. Synergistic anti-inflammatory effect of ILBI was evident, if applied additionally in combination with tocilizumab, in a patient with a therapy-resistant severe form of JIA and related subacute transient immunodeficiency.

## 1. Introduction

Juvenile idiopathic arthritis (JIA) is an inflammatory disorder of the connective tissues, characterized by joint swelling, pain, and tenderness. Overproduction of tumor necrosis factor alpha (TNF-*α*), interleukin-6 (IL-6), and interleukin-1 beta (IL-1*β*) is a well-known fact in JIA. Increased levels of these cytokines, both in serum and synovial fluid, induce the production of vascular endothelial factor, triggering angiogenesis in the affected joint. IL-6 is also considered responsible for osteoclast differentiation, followed by joint destruction and osteoporosis [1].

In the last decade, the development of biological agents that target these key inflammatory cytokines or their receptors brought new horizons in JIA treatment. Disease remission became a reachable goal [2]. However, few patients develop a severe chronic rheumatic type of the disease [3]. TNF-*α* inhibitors are the first-line biological agents used in JIA, if therapy with anti-inflammatory and disease-modifying antirheumatic drugs (DMARDs) proved to be inefficient. IL-6 receptor inhibition with Tocilizumab is a new second-line therapy for anti-TNF-*α* nonresponding patients, based on the blockade of IL-6-regulated signaling pathways [1, 2]. The main disadvantage of DMARDs and biologics is the temporary effect of immunosuppression [4].

Photobiostimulation could be able to modulate the immune system response. There are some studies that have shown the effect of intravenous laser blood irradiation (ILBI) in several immune-related diseases [5] and some studies of the authors with regard to ILBI in JIA patients [6], but there is no data at all about ILBI in association with Tocilizumab.

## 2. Patient and Method

A 16-year-old male was admitted in July 2011 to “St. Mary” Emergency Hospital for Children, Iasi, Romania, and was diagnosed with JIA—persistent oligoarthritis type. He reported swollenness of right ankle and left knee, pain, and walking difficulties.

Family history was remarkable for rheumatoid arthritis (mother) and type I diabetes mellitus (grandmother). His past medical history revealed scarlet fever (2004), right elbow fracture (2005), viral meningitis (2007), type A hepatitis (2009), and recurrent enteritis and infectious mononucleosis (2011).

Inflammation markers were increased: erythrocyte sedimentation rate (ESR) = 45 mm [*n* < 15 mm/1 h]; fibrinogen = 520 mg/dL [*n* = 200–400 mg/dL]; C-reactive protein (CRP) = 15.2 mg/dL [*n* < 6 mg/dL]. Both IgG and IgM antibodies for Epstein-Barr virus were positive. Rheumatoid factor, anti-streptolysin O antibodies, hepatitis B, hepatitis C, HIV type 1, and HIV type 2 antibodies were negative. Blood, urine, and stool cultures were negative.

He received treatment with NSAIDs, intra-articular Prednisolone (50 mg/large joint, every 3 months), a DMARD (Methotrexate, 20 mg/week), and physical therapy.

In the following six months, the patient had epidemic parotitis and several chest infections, most probable due to the immunosuppressant effect of Methotrexate. He recovered completely and continued to take his regular medication for JIA.

In February 2012, the patient returned to our clinic, evolving to severe oligoarthritis–extended type. Inflammation markers titers were higher that time (ESR = 78 mm/h, fibrinogen = 728 mg/dL, CRP = 162 mg/dL) and selective IgA (12 mg/dL, *n* = 70–400 mg/dL) and IgG (230 mg/dL, *n* = 700–1600 mg/dL) deficiencies were firstly detected. Levels of circulating immune complexes (104.5 *μ*g/mL) [*n* < 40 *μ*g/mL] and anti-cyclic citrullinated peptide antibodies (28.6 U/mL) [*n* < 15 U/mL] were increased. HLA-B27 test was negative. Bone marrow aspirate excluded malignancy.

On the top of current medication, an anti-TNF-*α* agent (Etanercept—0.4 mg/kg, twice a week) was introduced together with 5 daily IV injections of Prednisolone (30 mg/kg, max 1 g). The patient was discharged on Methotrexate, Etanercept, and oral corticosteroids in daily alternation with NSAIDs. A proton-pump inhibitor (PPI), Omeprazole (20 mg once daily), was associated to the anti-inflammatory medication to counterbalance its gastrointestinal side effects. Etanercept represents the first biological agent to be introduced in JIA in Romania, according to national guidelines, which are in accordance with the European guidelines [7].

In May 2012 the patient was readmitted to the hospital with an altered general status, prolonged intermittent pyrexia, violent pain, swelling, and important polyarticular dysfunctions (16 joints with active arthritis, 47 joints with limited range of motion). The diagnosis was revised to polyarthritis (FR negative) and acquired immunodeficiency. Etanercept was changed to an anti-IL-6 receptor inhibitor (Tocilizumab—8 mg/kg, every 2 weeks) due to the poor response to anti-TNF-*α* therapy, and opiate analgesics were introduced as required. Normally, anti-TNF-*α* therapy is revised after 3 months, and decision to change the biologic agent is made if the ongoing one is not controlling the disease.

In August 2012, the patient maintained almost the same diseases activity, objectified by ACR core set data [8]. While JIA was not controlled, IgA and IgG deficiencies were still present, and the patient did not respond to two biological agents from different classes of action (Etanercept, followed by Tocilizumab), the decision was made to try additional ILBI on the top of ongoing medication, as a last resort.

It is important to mention that, in Romania, Etanercept is the only medication for JIA which has the entire cost reimbursed by Romanian National Health Insurance House at present [7]. Tocilizumab is a new biologic that recently entered in our University Pediatric Clinic through a pilot program and is reserved to severe cases which do not respond to Etanercept.

We have clearly explained the ILBI method to the patient and the family; we informed them that there were cases where ILBI was applied in adults with immune disorders successfully without side effects, but still it has not been applied before in children or adolescents with JIA who simultaneously take Tocilizumab.

**Figure 1:**
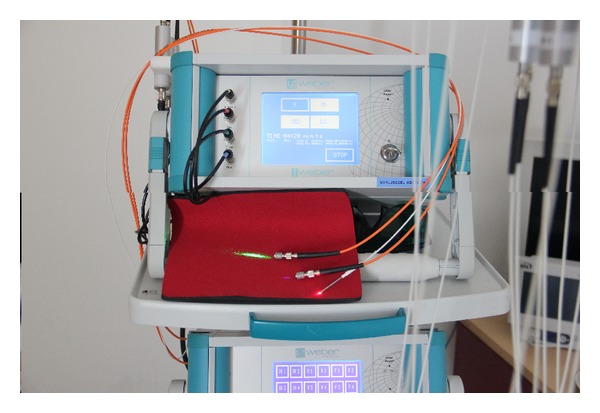
Weberneedle Endolaser. The following wavelengths were used: 630 nm (red), 536 nm (green), and 405 nm (violet).

With the involvement of all the authors, we managed to offer the patient, free of charge, the possibility of receiving intravenous laser blood irradiation (ILBI) using a Weberneedle Endolaser (weber medical, Lauenförde, Germany; see also Figure 1).

The patient and family consented, and on the top of ongoing therapy 30 ILBI sessions were given over 6 months.

ILBI protocol used 3 different wavelengths, administered to the patient in the following sequence: red radiation (630 nm) at the beginning of the session, followed by green radiation (536 nm) and blue (violet) radiation (405 nm) at the end. The reason for this sequence was to deliver an increasing amount of energy to the blood stream (because a blue (violet) photon has more energy than a red photon). The laser device delivered a 5 mW maximum output power in the continuous mode. The patient received 20 minutes of laser radiation for each wavelength, 60 minutes in total, per session.

The patient underwent 1 session daily and 10 successive sessions per month, repeated every 3 months, for 7 months long (30 sessions in total). To assess ILBI outcome, patient evaluation was performed before ILBI was started and at 3, 6, 9, and 12 months, respectively, after initiation of this therapeutic method (Table 1).

Laser radiation was given into a vein located at the cubital region, by a sterile optical fiber, which was passing through the lumen of a butterfly needle (size 21G). Proper skin disinfection was performed before the vein puncture. The needle and the external part of the optical fiber were immobilized to the patient forearm with adhesive tape. At the end of the session the needle and the catheter were extracted and a sterile swab was applied at the puncture site. Skin desensitization with local anesthetic cream was not necessary due to the patient's excellent compliance to vein puncture.

## 3. Results

First evaluation after ILBI revealed a good improvement in pain (35% less on visual scale), joint count (active arthritis,9 joints, limited movement,30 joints), clinical assessment, and ESR. The patient fulfilled the ACR Pediatric 50 response, and oral corticosteroids were excluded by a decreasing-dose protocol.

ACR Pediatric 30 (50, 70, 90, resp.) criteria, which we used in our evaluation, are defined as improvement of more or 30% (50%, 70%, 90%, resp.), in at least 3 of the 6 core set variables used to assess disease activity, with no more than 1 variable worsening by more or 30% [8].

After finishing all 30 ILBI sessions (6 months), the patient had an ACR Pediatric 70 response. Laboratory findings displayed normal IgA and IgG levels.

At 9 months from ILBI initiation (May 2013), the patient displayed an ACR Pediatric 90 response. Inflammation markers and immunoglobulin titers were normal. This promising outcome due to ILBI allowed the withdrawing of Methotrexate and NSAIDs, and patient continued only on Tocilizumab.

**Figure 2:**
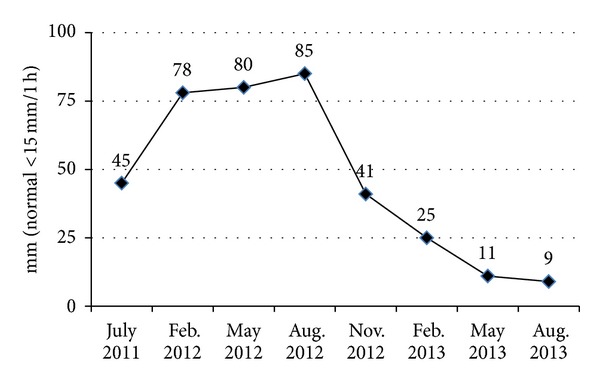
ESR evolution between July 2011 and August 2013.

At 12 months from ILBI initiation (August 2013), the patient continued only on Tocilizumab managing to maintain an ACR Pediatric 90 response, and the inflammation markers were normal. Another evaluation after another 3-month period, in November 2013, was not possible as the patient and his family moved abroad.  Figure 2 displays the evolution of ESR between July 2011 and August 2013.

## 4. Discussions

ILBI represented a therapeutic solution for a patient who was not responding to remissive treatment with DMARDs, steroids, and two biological agents targeting different inflammatory cytokines. Laser therapy is not a conventional method within the standard international treatment protocols for JIA [3].

ILBI was performed firstly in the former Soviet Union at the beginning of 1980s. At that time, the results were only published in Russian, and this therapeutic method remained mostly in a corner of shadow in Europe and the United States, up to 10 years ago [5].

In this case study, the therapeutic protocol was the same before and during the first 2 sets of ILBI sessions, consisting in Tocilizumab, Methotrexate, corticosteroids, and painkillers. The only difference was the additional laser irradiation. Thus, ILBI increased the effect of IL-6 receptor inhibition, and possibly triggered further immunomodulatory responses.

ESR evolution from July 2011 to August 2012 shows how the inflammatory response evolved and that it was not effectively controlled, even if classical DMARDs, corticosteroids, Etanercept, and Tocilizumab were introduced.  Figure 2 displays the shift produced in the patient's overall inflammatory response after the two sets of ILBI sessions. Even if we cannot fully explain all the phenomena al cellular level, the descending trend of this inflammation marker, after ILBI initiation, supports the immunomodulatory effect of low-level laser radiation.

The normalized ESR values and the ACR Pedi 90 response maintained for another 3-month interval, from May 2013 to August 2013, show that ILBI managed to have a beneficial anti-inflammatory and immunomodulatory intervention which shifted the clinical evolution of the patient into a positive direction. However, we cannot give information if ILBI would have required a periodic maintenance, as the patient had a very good clinical response, and only Tocilizumab was kept as single therapy. At the moment, we do not have data regarding ILBI administration for more than three consecutive sets of 10 sessions per month [6]. Unfortunately, there are no other data in the scientific literature regarding ILBI and Tocilizumab synergic administration that we could comment upon.

The first evaluation after 3 months from ILBI initiation, immediately after the second set of laser sessions, allowed the consultant doctor who was supervising the patient to exclude the sistemic corticosteroid therapy. Our local practice protocol places an emphasis on limiting the use of steroidal medication, due to its important side effects. An ACR Pedi 50 response was a clear indication for discontinuation of the corticosteroids, with gradual withdrawing. The tapering of the steroid dose was done during a 10-day interval to prevent steroid withdrawal symptoms. However, we cannot evaluate if there was another interaction between the laser radiation and corticosteroid therapy, except the overall anti-inflammatory synergistic effect.

Before starting ILBI and during the first month after ILBI initiation, the patient received opiate medication on an as-required basis (Codeine Phosphate, 15–30 mg per oral, up to three times a day, depending on the pain intensity). After that time, he remained on his monthly prescription which included, besides the remissive treatment, only NSAIDs as painkillers.

Photosensitization is a side effect that affects the skin of 1 to 3% of the patients receiving Methotrexate or NSAIDs, according to the manufacturers' official product information leaflet. This phenomenon appears when a “light sensitive” compound, like Methotrexate, is exposed mainly to UVA radiation. Surveying this possible side effect during ILBI was a priority for us. We also advised the patient to avoid direct sunlight and to use protective creams for the sun-exposed skin areas at all times, during Metotrexate and NSAIDs administration.

This patient did not report any photosensitization reactions. Any photosensitization reaction would have leaded to laser therapy discontinuation. It is important to mention that the laser device delivered only a 5 mW maximum output power in the continuous mode, and the blue radiation (405 nm) which is closer to the UVA spectrum lasted only for 20 minutes per session.

When ACR Pedi 90 response was obtained, after 9 months from ILBI initiation, Methotrexate and NSAIDs were discontinued. Methotrexate discontinuation was awaited due to its nontargeted immunosuppressant effect.

The transient immunodeficiency the patient encountered was most probably due to immunosuppressive treatment with Methotrexate. This was revealed by the often infectious diseases the patient developed and also by altered specific laboratory findings. The association between JIA and acquired immunodeficiency could represent a challenge, for both the doctor and the family of the affected child.

NSAIDs have a well-known renal toxicity, together with an increased risk for gastrointestinal (GI) and cardiovascular side effects, when given on a long-term basis [9]. To counteract the GI unwanted disturbances, a PPI was associated. Moreover, when the patient clinical status allowed, NSAIDs were withdrawn to eliminate their toxicity entirely, and the patient continued only on biologic medication. Regarding the interaction between NSAIDs and ILBI we needed to constantly evaluate for an unwanted reaction of photosensitization, which did not occur in this case study.

The patient and his family consented for starting ILBI, even if there is no data regarding its application in JIA patients who receive Tocilizumab. But the information with regard to its value in other immunological conditions [5] constituted a promising background.

The result obtained after 9 and 12 months from the initiation of laser therapy points to the immunomodulatory effect of ILBI.

There is data in the literature pointing to the effect of laser radiation in experimentally induced acute inflammation in rat lungs by decreasing the titers of proinflammatory cytokines, like TNF-*α* [10, 11] and IL-6 [10]. Aimbire et al. concluded that these effects are dose dependent [10] and Boschi et al. suggested that the decrease in IL-6 titers could even occur at lower laser energy-delivered doses, compared to the ones necessary for TNF-*α* titre reduction [11]. Bjordal et al. revealed that there is strong evidence, from 19 out of 22 placebo-controlled laboratory studies, regarding the reduction of proinflammatory cytokines (TNF-*α*, IL-6, and IFN-*γ*) in the laser-irradiated animal subjects [12].

Following the same pathway in which laser radiation decreases the levels of proinflammatory cytokines, Shiba et al. proved that Nd:YAG laser radiation can abolish the increase in IL-6 levels *in vitro*, in human pulp cells [13].

A different hypothesis, explaining the beneficial effect ILBI had in our JIA patient, who was also receiving an IL-6 receptor antagonist, could be that laser radiation increases the blocking effect of Tocilizumab on the IL-6-regulated inflammation pathways. This effect can be paralleled with the *in vitro* research done by Reale et al. on human monocytes, who displayed that infrared laser irradiation enhances IL-1 receptor antagonist [14].

According to Miyazawa et al. IL-1beta stimulated rheumatoid fibroblast-like synoviocytes (FLSs) produce IL-6 in a concentration- and time-dependent manner. In this study they demonstrated how the IL-6 promoter is transcriptionally regulated in rheumatoid FLSs in response to a physiologically relevant mediator of inflammation, IL-1beta [15]. So, inhibiting the IL-1 pathway with laser radiation [14] could reduce the production of IL-6, and Tocilizumab molecules might have “less” IL-6 molecules to compete with, at IL-6 receptor level.

The influence on the IL-6 pathway by IL-1 activity was also demonstrated by Radtke et al. who showed that p38 mitogen-activated protein kinases, which are previously activated by IL-1beta, impair IL-6-induced signalling through phosphorylation of the common cytokine receptor subunit gp130 and determine its subsequent internalisation and degradation [16]. Following this rationale, we could suppose that laser radiation could have determined a membrane IL-6 receptor downregulation. IL-6 receptors exit in membrane (mIL-6R) and soluble (sIL-6R) forms. The later appears via proteolytic cleavage of the mIL-6R, which leads to the generation sIL-6R [17].

Downregulating mIL-6R would also decrease the titres of sIL-6R, and this would mean a greater probability for a Tocilizumab molecule to bind to either a mIL-6R or a sIL-6R in the competition with the serum IL-6 molecules. The result would be a more efficient IL-6 proinflammatory blockade.

A third explanation for the positive immunomodulatory effect ILBI could be the capacity of laser radiation to balance the proinflammatory status by increasing the anti-inflammatory cytokines. Animal studies revealed increased IL-10 after low-level laser stimulation [18].

Because, at the end of the ILBI sessions, the patient not only had a favorable clinical response, but also displayed normal IgA and IgG levels, we assume that laser therapy acted on several pathways in modulating the immune response.

There is strong evidence for ILBI efficacy, demonstrated by the favourable change in the evolution of the patient. Before administering ILBI, the patient was already on an unfavourable evolution for 3 months, even if he was receiving Tocilizumab. ILBI was applied on top of the existing treatment protocol, and everything else remained unchanged for another 3 months. Thus we consider that the improvement in the patient condition and the ability to start controlling the disease is not a statistical variation in the population and the disease group, regardless the use of ILBI.

Our case study opens the way for further studies on statistically significant groups of patients, in order to verify the promising results we encountered with ILBI and an IL-6 receptor antagonist in JIA.

## 5. Conclusions

Synergistic anti-inflammatory effect of intravenous laser blood irradiation was evident, if applied in combination with Tocilizumab, in a patient with a therapy-resistant severe form of juvenile idiopathic arthritis and related subacute transient immunodeficiency.

Further studies are necessary to verify this association efficacy, which we found for the first time in the scientific literature.

## Figures and Tables

**Table 1:** Timeline for ILBI set of sessions and ACR Pedi evaluation.

Month number	1	2	3	4	5	6	7	8	9	10	11	12	13
ILBI set of 10 sessions	S1			S2			S3						
Evaluation	E0			E1			E2			E3			E4

S1: first set of ILBI sessions; S2: second set of ILBI sessions; S3: third set of ILBI sessions; E0: first evaluation before ILBI treatment started; E1–E4: further evaluations.
